# Research of influence and mechanism of combining exercise with diet control on a model of lipid metabolism rat induced by high fat diet

**DOI:** 10.1186/1476-511X-12-21

**Published:** 2013-02-20

**Authors:** Chen Shaodong, Zhou Haihong, Lin Manting, Li Guohui, Zhao Zhengxiao, Zhang YM

**Affiliations:** 1 Medical College of Xiamen University, 361005, Xiamen, China

**Keywords:** Lipid metabolism disorder, Rat, Intervention, Gastrointestinal hormones

## Abstract

**Objective:**

To investigate the influence and mechanism of combining exercise with diet control on a model of lipid metabolism rat induced by high fat diet.

**Methods:**

Twenty-four male Wistar rats were randomly divided into 3 groups of 8: normal, model and intervention. The model group and intervention group were fed with high fat diet, while the normal group received basal feed. From day 1, the intervention group was randomly given interventions such as swimming exercise and dietary restriction. The interventions duration were 28 days. At the end of the experiment, the levels of rats’ body weight and liver weight were detected, the serum levels of total cholesterol (TC), high density lipoprotein cholesterol (HDL-C), low density lipoprotein cholesterol (LDL-C) and hepatic triglyceride content (TG) were detected by using biochemical assay, serum level of gastrin (GAS), motilin (MTL) were assayed by the enzyme linked immunosorbent assay (ELISA).

**Results:**

Compared with the level of body weight and liver weight in the normal rats, body weight and liver weight in the rat of the model group were significantly increase (P<0.05 or P<0.01). Plasma concentrations of TC, LDL-C and hepatic TG in the model group were significantly increased compared with those in the normal group (P<0.05 or P<0.01). The contents of GAS, MTL, HDL-C in the model rats’plasma were significantly reduced compared with those of the normal group (P<0.05 or P<0.01). Compared with those in the model group, rats’ body weight, liver weight, serum TC, LDL-C, and TG content of liver in the intervention group decreased significantly (P<0.05 or P<0.01). Meanwhile, serum content of GAS, MTL, HDL-C were significantly improved in the intervention rats compared to the model group.

**Conclusion:**

The action of combining exercise with diet control for lipid metabolism disorder might be related to regulation of GAS, MTL and other gastrointestinal hormones.

## Introduction

Lipid metabolism disorder refers to a disruption of lipid metabolism with exceeding serum levels of total cholesterol (TC), triglyceride (TG), low density lipoprotein cholesterol (LDL-C), and/or lower level of high density lipoprotein cholesterol (HDL-C). Serum levels of lipids and lipoprotein lipids are among the most potent and best substantiated risk factors for atherosclerotic diseases, particularly coronary heart disease (CHD) [1,2]. Therefore, it is very important to strengthen the prevention and cure of lipid metabolism disorder. In able-bodied individuals, exercise programs are important in the prevention and control of lipid metabolism disorders. Systematic reviews have also identified strong evidence that exercise and dietary restriction can improve lipoprotein levels in overweight able-bodied individuals [3]. But mechanism of the intervention of combining exercise with diet control remains to be further defined. It is now recognized that lipid metabolism is regulated by the reciprocal actions of a group of anorexigenic peptides, which include leptin, insulin, cholecystokinin, peptide YY and glucagon-like peptide. The majority of these hormones are secreted by endocrine cells scattered throughout the gastrointestinal tract [4]. While many studies reveal that gastrointestinal hormones play a notable role in regulating energy intake [5,6]. In this paper, we will focus on the underlying mechanisms of the intervention of combining exercise with diet control on the lipid metabolism disorder.

## Methods

### Reagents

TG kit, TC kit, HDL-C kit and LDL-C kit were purchased from Nanjing Jiancheng Biotechnology Co.,Ltd. (Nanjing, China). Gastrin(GAS) ELISA kit and motilin (MTL) ELISA kit were obtained from R&D Co.,Ltd.(USA).

High fat diet include 83.25% basal feed, 10% lard, 1.5% Clesterol, 0.2% NaTDC, 5% Sugar and 0.05% Propylthiouracil.

### Animal grouping

Twenty-four male Wistar rats (175±7 g) provided by the Experimental Animal Center of Medical College of Xiamen University (Xiamen, China), Animal License Key:SCXK (Hu) 2007_0005) were used. The use of animals in this study was approved by the Ethics Committee of Xiamen University (protocol XMULAC20120020).

Rats were randomly divided into 3 groups of 8: normal, model and intervention. From the first day of experiment, animals in the normal group were given basal feed, while rats in the model group and intervention group were fed with high faty diet for 28 days. In the meantime, rats in the intervention group were given different interventions randomly such as swimming exercise and dietary restrictions everyday. Time of swimming exercise was 30 minutes, deepth of water was 55 centimeter and temperature of water was 25°C. The amount of food for rats in the intervention group was restricted to 50% of the model group.

### Determination of level of body weight and liver weight

Rats were weighted at the day 1 and day 28 of the experiment. At the day 28, rats were injected with 10% chloral hydrate (0.36 mL/100 g body weight intraperitoneal injection). After the injection of anesthesia, rats underwent laparotomy. Liver in rats were weighted.

### Determination of serum TC, LDL-C and HDL-C Content

Blood (5 ml) was draw from the abdominal aortic into a tube, and was centrifuged at 10,000×g for 10 min at 4°C. The supernatant was then used to measure the content of TC, LDL-C and HDL-C in serum by atomic absorption spectroscopy.

### Determination of the concentration of Plasma GAS, MTL

Plasma stored in −20°C at room temperature was allowed to thaw. It was then centrifuged at at 10,000×g for 10 min at 4°C. The supernatant was removed, and the content of GAS and MTL plasma was measured using the enzyme linked immunosorbent assay (ELISA).

### Determination of hepatic TG content

For the measurement of TG in liver tissue, frozen liver tissues were homogenized in PBS, and methanol was added to the lysate. The lipids were extracted by the method of Bligh and Dyer [7], and triglyceride content was measured using a TG kit.

### Statistical analysis

Data are expressed as mean±standard deviation. Data were analyzed using Statistical Package for Social Sciences v11.0 software (SPSS, Chicago, IL, USA). One -way analysis of variance (ANOVA) followed by q test were used to determine significant differences among the 3 groups. P<0.05 was considered significant.

## Results

### Comparison of levels of body weight and liver weight

On day 1 of the experiment, body weight in each group were not significantly different (P>0.05). On day 28, compared with the normal group, the body weight and liver weight of the model group were significantly increased (P<0.05, P<0.01). This finding suggested that raising the rats in high fat diet could successfully establish a model of lipid metabolism disorder. Compared with the model group, body weight and liver weight of the intervention group had remarkably decreased (P<0.05, P<0.01). This finding suggested that intervention had effects in improving disturbance of lipid metabolism (Table 1).

**Table 1:** **Comparison of body weight and liver weight (**$\bar{x}\pm s$**)**

**Group**	**n**	**Body weight (g)**	**Liver weight(g)**
normal	8	250.6±40.2	8.97±0.24
model	8	314.6±39.3^*^	13.33±0.74^**^
intervention	8	265.8±20.6^#^	10.93±0.5^##^

Notes: ^*^P<0.05, ^**^P<0.01, compared with the normal group;

^#^P<0.05,^##^P<0.01, compared with the model group.

### Comparison of serum levels of TC, LDL-C,HDL-C and hepatic level of TG

Compared with the content of TC, LDL-C and HDL-C in the serum of rats of the normal group (4.00±0.73 mmol/L, 2.28±0.89 mmol/L and 0.57±0.14 mmol/L respectively), serum TC, LDL-C levels in model rats increased significantly to 5.98±1.07 mmol/L, 4.03±0.96 mmol/L and the HDL-C content was significantly reduced to 0.24±0.03 mmol/L (P<0.05, P<0.01). In the meantime, compared with the level of hepatic TG in the rats of the normal group (1.42±0.51 mmol/L), the content of hepatic TG in the rats of the model group was significantly increased to 3.31±0.74 mmol/L (P<0.01). This finding suggested that lipid metabolism disorder in a model of rats.

Compared with the model group, the intervention group had a regulatory role in lipid metabolism. Serum levels of TC, LDL-C and hepatic TG content in the rats of the intervention group were lower (4.40±0.64 mmol/L,2.97±0.68 mmol/L and 2.44±0.19 mmol/L), whereas HDL-C content increased significantly to 0.33±0.06 mmol/L (P<0.05, P<0.01, Table 2).

**Table 2:** **Comparison of serum levels of TC, LDL-C,HDL-C and hepatic level of TG(**$\bar{x}\pm s$**)**

**Group**	**n**	**Hepatic TG(mmol/L)**	**Serum TC(mmol/L)**	**Serum LDL-C(mmol/L)**	**Serum HDL-C(mmol/L)**
normal	8	1.42±0.51	4.00±0.73	2.28±0.89	0.57±0.14
model	8	3.31±0.74^**^	5.98±1.07^**^	4.03±0.96^*^	0.24±0.03^**^
intervention	8	2.44±0.19^#^	4.40±0.64^##^	2.97±0.68^#^	0.33±0.06^#^

Notes: ^*^P<0.05, ^**^P<0.01, compared with the normal group;

^#^P<0.05,^##^P<0.01, compared with the model group.

### Comparison of the concentration of plasma GAS, MTL

The plasma concentrations of GAS and MTL of rats in the model group were 0.183±0.006 ng/mL and 0.224±0.022 ng/mL, respectively. Compared with the respective values in the normal group, 0.209±0.010 ng/mL and 0.279±0.033 ng/mL, they were significantly decreased (P<0.05). These findings suggested there is a change in gastrointestinal hormones for lipid metabolism disorder.

The plasma concentrations of GAS and MTL of the intervention group was significantly higher than those in the model group (0.206±0.008 ng/mL and 0.260±0.026 ng/mL, respectively, P<0.05, Table 3). This finding suggested that intervention improved gastrointestinal hormones dysfunction.

**Table 3:** **Comparison of the concentration of plasma GAS, MTL(**$\bar{x}\pm s$**)**

**Group**	**n**	**GAS(ng/mL)**	**MTL(ng/mL)**
normal	8	0.209±0.010	0.279±0.033
model	8	0.183±0.006^*^	0.224±0.022^*^
intervention	8	0.206±0.008^#^	0.260±0.026^#^

Notes: ^*^P<0.05, compared with the normal group;

^#^P<0.05, compared with the model group.

### Discuss

Modern day lifestyle and diet dramatically increase the threat of lipid metabolism disorder. This metabolic disorders has been consistently linked to the imbalance between energy consumption and expenditure. Growing evidence suggests that faulty regulation of lipid metabolism promotes metabolic diseases [8]. As we know, the exercise and diet control are the best way to control the lipid metabolism disorder.

In this study, the model of lipid metabolism disorder was built successfully induced by high fat diet rats. The level of body weight, liver weight, plasma concentrations of TC, LDL-C and hepatic TG were significantly increased,while plasma concentrations of HDL-C was significantly reduced. Combining exercise with diety restriction has function to improve the above index. It is hypothesized by Richter EA that exercise-induced increases in AMP-activated protein kinase (AMPK), an enzyme that, among many other functions, stimulates glucose uptake and fatty acid oxidation while decreasing lipid synthesis, may explain the observed associations between exercise and reduced metabolic disease [9-12].

In recently, the role of gastrointestinal hormones play in the regulation of lipid metabolism become more and more important. Gastrointestinal hormones has function to optimize the process of digestion and absorption of nutrients by the gut [13]. By altering the rate at which nutrients are delivered to compartments of the alimentary canal, the control of food intake arguably constitutes another point at which intervention may promote efficient digestion and nutrient uptake [14].

Motilin is one of gastrointestinal hormones, a 22-amino-acid peptide synthesized from endocrine cells of the duodeno-jejunal mucosa [15], interacts directly with its receptor, induces smooth muscle contraction and improves peristalsis in the small intestine [16,17]. Motilin regulates the interdigestive migrating contractions (IMC), the fasted motor pattern in the gastrointestinal (GI) tract [18]. Motilin plasma levels increase cyclically every 90–120 minutes during the interdigestive fasting period, and this cyclical release of motilin disappears after ingestion of a meal. These cyclical peaks of plasma motilin are synchronized to strong peristaltic contractions initiated from the stomach and migrating to the duodenum and small intestine. This pattern of migrating waves is known as the phase III contraction of IMC. In the present study, the serum motilin level was significantly lower in model rats than in normal rats and significantly higher after intervention with combining exercise with diet control. It is reported in the study of Miegueu P, motilin may directly influence adipocyte functions by controlling energy storage [19].

Gastrin was subsequently shown to be secreted from neuroendocrine G cells which are principally located in the antrum of the stomach. The gastrin gene is located on the long arm of chromosome 17 and encodes a 101 amino acid polypeptide, preprogastrin. This gene product is subjected to a series of post translational modifications which result in the synthesis of a number of biologically active peptides [20]. Gastrin normally regulates gastric acid secretion by stimulating the proliferation of enterochromaffin-like cells and the release of histamine [21,22]. The researches about effect of gastrin on sphincter od Oddi are rare. But in the research of Saqui-Salces M, results show that components of food (fat) are sensed by antral cilia on endocrine cells, which modulates gastrin secretion and gastric acidity [23]. In our study, the content of serum GAS in rats of the model group decreased significally compared with those of normal group, while significantly increase after intervention.

In the research, it showed us gastrointestinal hormones play a critical role in the lipid metabolism disorder and intervention has function to improve the level of serum of GAS and MTL in the model rats.

## Competing interests

The authors declare that they have no competing interests.

## Authors’ contributions

CS and ZH carried out the animal experiment studies and drafted the manuscript. LM, LG, ZZ and ZYM participated in the molding process and carried out the detection index testing. All authors read and approved the final manuscript.
